# Magnitude and effects of diabetes mellitus, malnutrition and HIV comorbidities on the management and outcomes of paediatric tuberculosis in Dar es Salaam and Pwani, Tanzania: a mixed-methods protocol

**DOI:** 10.1136/bmjopen-2026-120133

**Published:** 2026-10-29

**Authors:** Theresia William Mbuli, Blandina Mmbaga, Kaushik Ramaiya, Sayoki G Mfinanga

**Affiliations:** 1 National Institute for Medical Reseach, Muhimbili, Dar es Salaam, Tanzania, United Republic of; 2 Kilimanjaro Christian Medical University College, Moshi, Tanzania, United Republic of; 3 Clinical Research, National Institute for Medical Research Muhimbili Research Centre, Dar es Salaam, Tanzania, United Republic of; 4 Kilimanjaro Clinical Research Institute, Kilimanjaro Clinical Research Institute, Moshi Urban, Kilimanjaro Region, Tanzania, United Republic of; 5 Internal Medicine, Shree Hindu Mandal Hospital, Dar es Salaam, Tanzania, United Republic of; 6 Public Health, Kampala International University - Dar es Salaam College, Dar es Salaam, Tanzania, United Republic of

**Keywords:** Epidemiology, Tuberculosis, Treatment Outcome, HIV & AIDS, Diabetes Mellitus, Type 1, NUTRITION & DIETETICS

## Abstract

**Abstract:**

**Introduction:**

Tuberculosis (TB) remains among the leading causes of morbidity and mortality globally. Although children account for a substantial proportion of the TB burden, paediatric TB is frequently underdiagnosed because of limited bacteriological confirmation and non-specific clinical presentations. In many high-burden settings, including Tanzania, children with TB frequently present with comorbid conditions such as malnutrition, HIV infection and, increasingly, diabetes mellitus (DM). These comorbidities may influence susceptibility to infection, disease progression and treatment outcomes. Despite the increasing recognition of these interactions, limited research has investigated their combined magnitude and impact among children with TB in Tanzania. This study aims to determine the magnitude and effect of DM, malnutrition and HIV among children diagnosed with TB in the Dar es Salaam and Pwani regions.

**Methods and analysis:**

This mixed-methods study will be conducted in 40 health facilities in Dar es Salaam and Pwani regions and is nested within the OPTIC-TB project. Quantitative components will include a cross-sectional analysis estimating the prevalence of DM, malnutrition and HIV among children with TB and a prospective cohort analysis evaluating treatment outcomes. Diagnostic delay and health-service utilisation will also be assessed. Data will be collected through structured caregiver questionnaires, anthropometric measurements, laboratory investigations and review of clinical records. Regression models will be used to assess associations between comorbidities and TB treatment outcomes while accounting for prespecified confounders and facility clustering. Qualitative interviews with caregivers and healthcare providers will explore barriers to and facilitators of paediatric TB diagnosis and management.

**Ethics and dissemination:**

Ethical approval for the parent OPTIC-TB trial was granted by the National Health Research Ethics Committee of the National Institute for Medical Research (NIMR), Tanzania (Ref. NIMR/HQ/R.8a/Vol.IX/4754; extension Ref. NIMR/HQ/R.8c/Vol.I/3195, valid until 28 February 2027). This nested study was approved by the Kilimanjaro Christian Medical University College (KCMC) University Research Ethics Review Committee (Research Proposal No. 1617; Ethical Clearance Certificate No. 2807, valid until 5 March 2027). Written informed consent will be obtained from caregivers and assent from eligible children. Findings will be disseminated through peer-reviewed publications, scientific meetings and stakeholder engagement.

**Registration number (Main OPTIC-TB trial):**

PACTR202501677523721.

**STRENGTHS AND LIMITATIONS OF THIS STUDY**

- The integrated mixed-methods design combines cross-sectional prevalence and service-utilisation analyses, prospective follow-up of treatment outcomes and qualitative interviews.
- Nesting within the multicentre OPTIC-TB platform enables standardised recruitment and access to routine diagnostic and clinical data across 40 facilities.
- The facility-based sampling strategy may under-represent children with tuberculosis (TB) who do not reach participating health facilities.
- The cross-sectional analyses cannot establish temporal order or causality, and TB-related weight loss or transient stress hyperglycaemia may introduce reverse causality.
- Restriction to children aged 0–10 years inherited from the parent OPTIC-TB protocol limits generalisability to adolescents.

## Introduction

 Tuberculosis (TB) remains one of the leading infectious causes of morbidity and mortality worldwide. The WHO estimated that 10.8 million people developed TB in 2023, including approximately 1.25 million children and young adolescents.^1^ Despite this burden, paediatric TB remains underdiagnosed and under-reported. Diagnostic difficulties arise from the paucibacillary nature of disease in children, non-specific clinical presentations, difficulties obtaining respiratory specimens and limited bacteriological confirmation.^2–4^ Consequently, many children are diagnosed using a combination of clinical, radiological and epidemiological evidence, which may contribute to delayed treatment initiation and uncertainty in burden estimates.^2–4^

Malnutrition is an important determinant of susceptibility to infection among children. Undernutrition impairs innate and adaptive immune responses, including T-cell function and macrophage activity required to control Mycobacterium tuberculosis.^5^ Low body mass index is associated with increased TB incidence,^6^ while malnutrition in children with TB has been associated with severe disease, delayed recovery and unfavourable treatment outcomes.^7^

HIV infection is another critical determinant of TB risk and clinical severity. Children living with HIV have an increased risk of TB disease, extrapulmonary or disseminated disease, diagnostic difficulty and mortality.^8^ The dual burden is particularly important in sub-Saharan Africa, where most paediatric HIV infections occur.^9^ Coinfection further complicates management because of overlapping clinical features, treatment interactions and the need to coordinate antiretroviral and anti-TB therapy.^8^

In addition to these established comorbidities, diabetes mellitus (DM) is important but poorly characterised in paediatric TB. Most evidence linking DM to TB comes from adults, in whom DM increases the risk of active TB and is associated with poorer treatment outcomes.^10–13^ Type 1 DM can occur throughout childhood, whereas type 2 DM is increasingly recognised in young people but remains less common in children aged 10 years or younger.^14^ Paediatric evidence from sub-Saharan Africa is limited; however, a study among children and young people with type 1 DM in Dar es Salaam identified a measurable burden of latent TB infection.^15^ The present study therefore does not assume that DM is common in young children; it will estimate its prevalence and distinguish established or newly confirmed DM from transient hyperglycaemia associated with acute TB illness.

The coexistence of malnutrition, HIV and DM may influence TB management and outcomes through overlapping immunological, clinical and health-service pathways.^5 8 10–13^ However, most studies have examined individual conditions rather than their combined burden, leaving limited evidence on their joint effects in children in high-burden settings.

Understanding the magnitude and effects of comorbidities among children with TB is essential for improving integrated clinical management strategies and strengthening TB control programmes. Identifying the prevalence of conditions such as malnutrition, HIV infection and DM among paediatric TB patients can inform screening practices and guide targeted interventions aimed at improving treatment outcomes. Furthermore, examining diagnostic delays and health-system barriers may provide insights into opportunities for improving early detection and care delivery.

### Conceptual framework

The revised conceptual framework distinguishes exposures, confounders, mediators and outcomes. Baseline comorbidities (malnutrition, HIV and confirmed DM) are the principal exposures. Prespecified potential confounders include age, sex, socioeconomic position, region, health facility, TB site and severity, diagnostic category and OPTIC-TB trial arm. Diagnostic delay and health-service utilisation may act as intermediate pathways between contextual determinants and treatment outcomes and will not automatically be adjusted for when estimating total effects. TB itself may also cause weight loss and transient stress hyperglycaemia; these reverse pathways are shown explicitly and will be addressed through cautious interpretation, temporal classification, confirmatory glucose testing and sensitivity analyses. The cross-sectional component will estimate prevalence and associations only, whereas the prospective cohort will establish that baseline comorbidity measurement precedes treatment outcomes. Figure 1 summarises these pathways.

**Figure 1:**
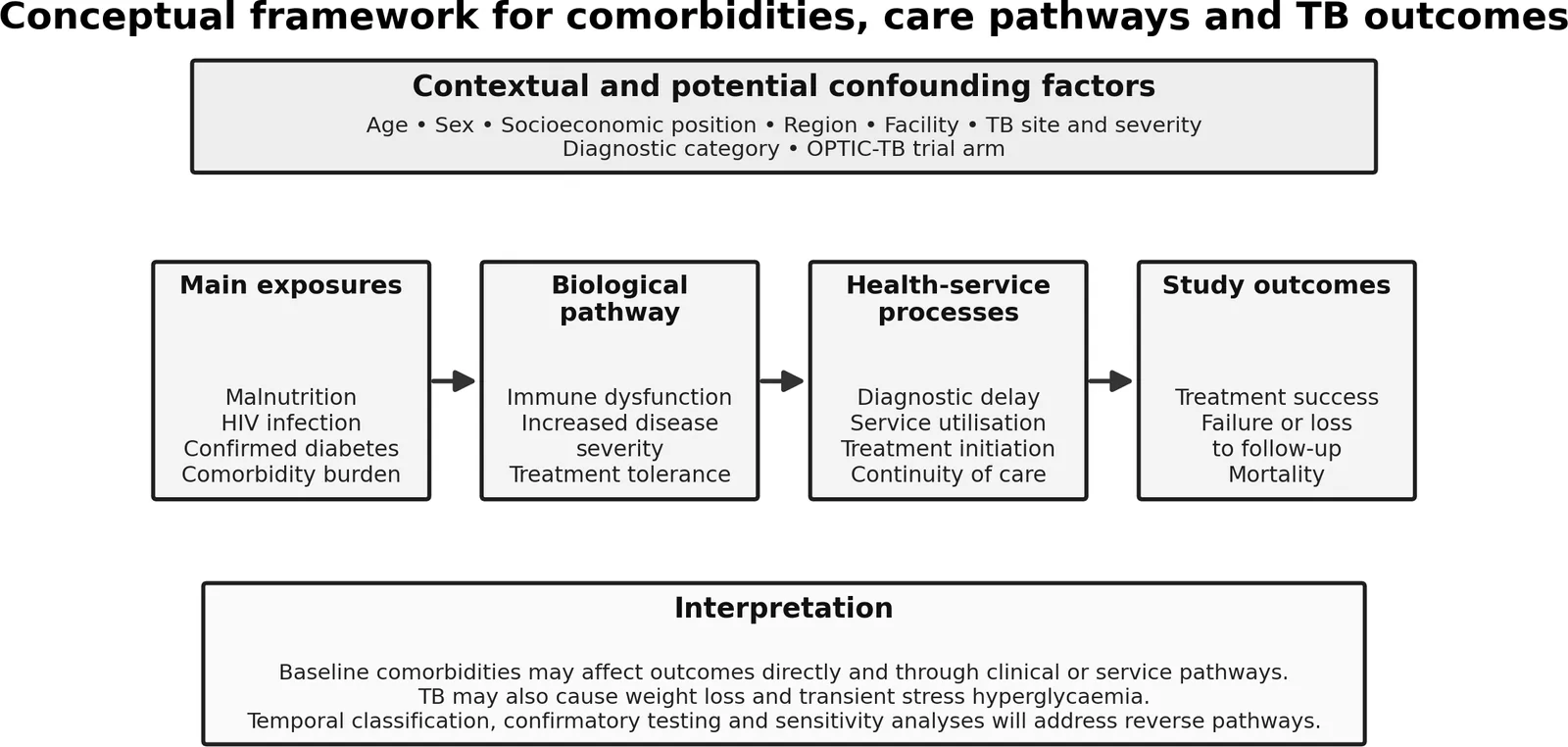
Conceptual framework for comorbidities, care pathways and treatment outcomes among children with tuberculosis. TB, tuberculosis.

### Study objectives

#### Primary objective

To determine the magnitude and effects of DM, malnutrition and HIV comorbidity on the management and treatment outcomes of paediatric TB.

#### Secondary objectives

1. To estimate the prevalence of DM, malnutrition and HIV among children diagnosed with TB and identify associated factors.
2. To assess the associations between these comorbidities and TB treatment outcomes.
3. To evaluate diagnostic delays among children with TB and examine whether these delays vary by comorbidity status.
4. To assess health service utilisation patterns among children with TB, including care-seeking pathways and the use of TB-related services, by comorbidity status.
5. To explore the perspectives of caregivers and healthcare providers on paediatric TB diagnosis, service utilisation and management.

## Methods and analysis

### Study design

This study will use a mixed observational design combining cross-sectional and prospective cohort components nested within the OPTIC-TB implementation trial (figure 2). All eligible children diagnosed with TB during the study period will be enrolled at baseline. Cross-sectional analyses will estimate the prevalence of DM, malnutrition and HIV and will examine diagnostic delay and health-service utilisation. The same participants will then be followed prospectively throughout TB treatment to determine treatment outcomes and assess whether outcomes differ according to baseline comorbidity status and comorbidity burden (zero, one, two or three conditions). This integrated design addresses multiple objectives within one population while reducing duplication and participant burden.

**Figure 2:**
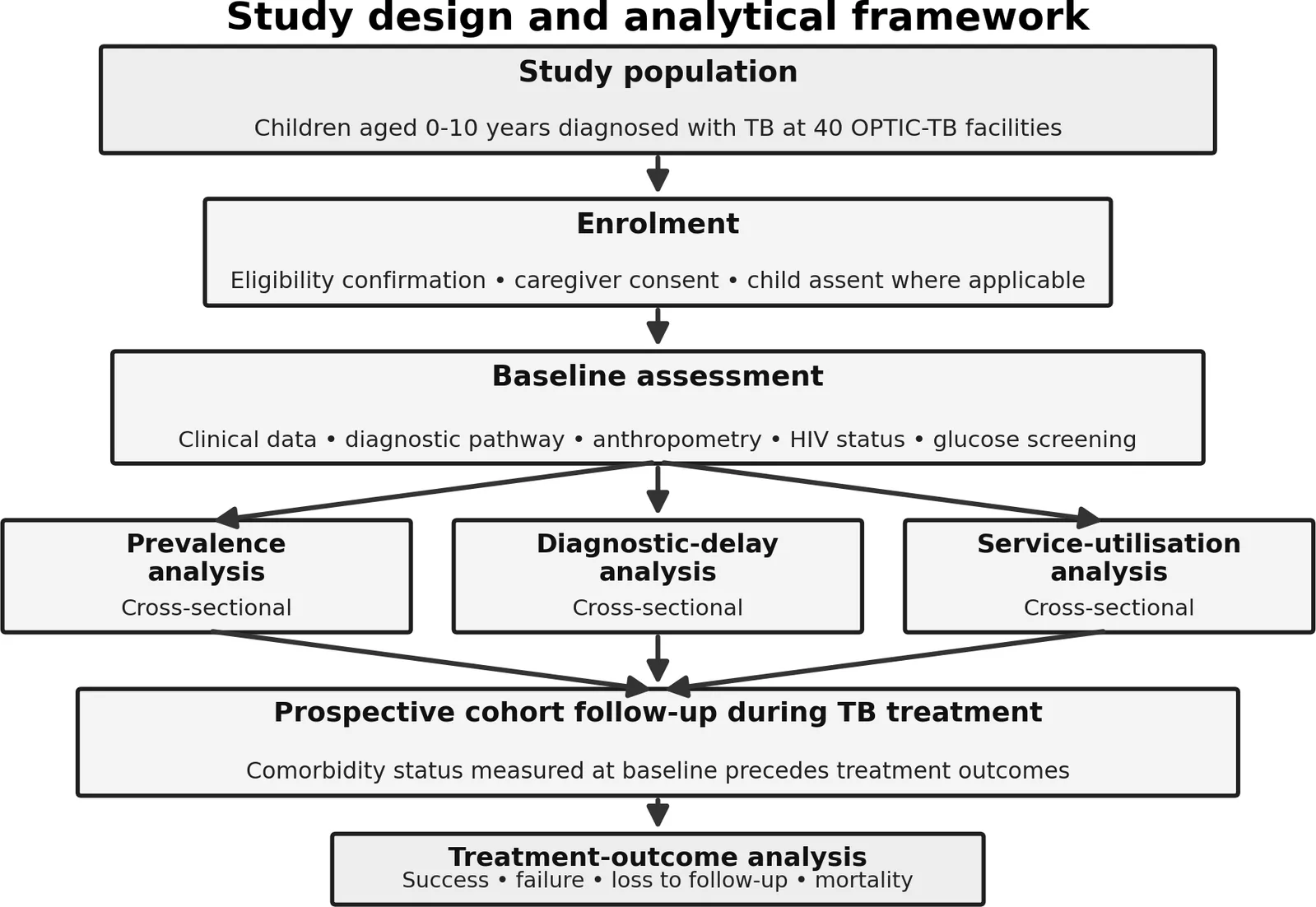
Study design and analytical framework. TB, tuberculosis.

### Study setting

The study will be conducted in 40 health facilities participating in the parent OPTIC-TB project in Dar es Salaam and Pwani regions, Tanzania. These regions represent urban, peri-urban and rural populations and contribute substantially to the national TB burden. Dar es Salaam, Tanzania’s largest city, reports a large number of TB cases annually and serves as an important referral hub for TB diagnosis and treatment. Pwani Region surrounds Dar es Salaam and includes both semi-urban and rural populations.

The participating facilities include regional referral hospitals, district hospitals, health centres and dispensaries providing paediatric TB diagnostic and treatment services. TB diagnostic services include clinical evaluation, radiological assessment and laboratory testing in accordance with national TB programme guidelines. The 40 facilities comprise 20 intervention and 20 control sites.

The intervention sites are Amana Regional Hospital, Chanika Health Centre, Chalinze Health Centre, Irene Kilimahewa Health Centre, Kirege Health Centre, Kigamboni Health Centre, Kigamboni Municipal Council District Hospital, Magomeni Health Centre, Mkoani Health Centre, Mkuranga District Hospital, Mnazi Mmoja District Hospital, Mwananyamala Regional Hospital, Mzinga Dispensary, Tabata A Dispensary, Tambukareli Dispensary, Tandale Health Centre, Temeke Regional Hospital, Tumaini Mission Health Centre, Tumbi Regional Referral Hospital and Vingunguti Dispensary.

The control sites are Bagamoyo District Hospital, Buguruni Health Centre, Bunju Health Centre, Charambe Dispensary, Kairuki, Kibiti, Kigogo, Kimbiji Health Centre, Kisarawe District Hospital, Kivule District Hospital, Madona Health Centre, Mbagala Rangi Tatu, Mbande, Mbezi Health Centre, Mlandizi Health Centre, Msoga District Hospital, Sinza Palestina, St. Vincent Health Centre, Tegeta Mission Dispensary and Ubungo District Hospital.

Together, these facilities represent diverse levels of healthcare delivery and urban, peri-urban and rural service settings across Dar es Salaam and Pwani regions.

### Study population

The study population will consist of children aged 10 years or younger who are diagnosed with TB and receive treatment at participating health facilities during the study period. The upper age limit is inherited from the parent OPTIC-TB protocol, whose diagnostic pathway, recruitment frame and paediatric assessment tools were designed for children aged 0–10 years. Retaining this limit preserves consistency with the parent study and reduces clinical heterogeneity associated with adolescent TB. However, it limits generalisability to adolescents, among whom TB and type 2 DM are increasingly important; this is acknowledged as a limitation and adolescent-specific research is recommended.

Children will be identified through TB registries, outpatient departments and paediatric wards in the participating facilities. Caregivers accompanying eligible children will be approached by trained research staff and invited to participate in the study.

### Eligibility criteria

Children will be eligible for inclusion if they are aged 10 years or younger and have bacteriologically confirmed or clinically diagnosed TB according to the Tanzania National Tuberculosis and Leprosy Programme algorithm. Bacteriologically confirmed TB will be defined by detection of Mycobacterium tuberculosis using Xpert MTB/RIF or Xpert Ultra, smear microscopy or mycobacterial culture where available, using respiratory or appropriate extrapulmonary specimens. Clinically diagnosed TB will be based on the national paediatric algorithm, incorporating symptoms and signs, TB contact history, tuberculin skin testing where available, chest radiography or other imaging, response to appropriate non-TB treatment and clinician judgement. The diagnostic tests performed, specimen type, result and basis of diagnosis will be recorded for each child, allowing site-level differences in diagnostic availability to be described. Children must be receiving TB treatment at a participating facility and have a parent or legal guardian able to provide written informed consent. Exclusion criteria remain incomplete key baseline data, caregiver refusal or withdrawal or transfer before baseline procedures.

### Sample size determination

Sample size calculations were conducted for four quantitative objectives: prevalence of comorbidities, diagnostic delay, service utilisation and treatment outcomes. Because the same participants will contribute to these analyses, the largest calculated sample size was selected. For prevalence estimation, Cochran’s single-proportion formula was used.^16 17^ Planning values included malnutrition prevalence of approximately 30%, HIV prevalence of 8%–24% and an exploratory DM range of 2%–10%; the DM range reflects sparse paediatric TB-DM evidence and was not treated as a local prevalence estimate.^1 6 14 15 18^ The prevalence objective required 379 participants after allowing for 15% attrition. Diagnostic-delay calculations compared two means with 80% power, 95% confidence and a SD of 10 days, informed by previous delay reviews;^19 20^ the conservative scenario required 302 participants after attrition. Service-utilisation calculations compared two proportions using published evidence on barriers to paediatric TB services^17 19 21^ and required 212 participants after attrition. Treatment-outcome calculations compared four comorbidity-burden strata and required 235 participants, informed by reported childhood TB outcomes.^1 7^ The study will therefore enrol at least 379 children. The assumptions, effect or precision targets and resulting sample sizes are summarised in table 1.

**Table 1:** Summary of sample-size assumptions for quantitative objectives

Quantitative objective	Comparison/parameter	Key assumptions	Effect/precision target	Power/confidence	Attrition	Required N
Prevalence of comorbidities	Single proportion	Highest expected prevalence: malnutrition 30%; DM 2%–10%; HIV 8%–24%	5% absolute precision	95% CI	15%	379
Diagnostic delay	Two independent means	SD 10 days; scenarios: 10 vs 13.5, 15 vs 30, 20 vs 40 days	Detect difference of 3.5–20 days	80% power; 95% confidence	15%	302
Service utilisation	Two proportions	60%–80% without comorbidity vs 30%–60% with comorbidity	Moderate comparison scenario	80% power; 95% confidence	15%	212
Treatment outcomes	Four comorbidity-burden strata	Treatment success 80%–90% with no comorbidity vs 35%–55% with multiple comorbidities	Approximately 50 participants per stratum	80% power; 95% confidence	15%	235
Unified study sample	Largest required sample	Based on prevalence objective	Ensures coverage of all quantitative analyses	–	Included	379

### Sampling strategy

Participants will be systematically recruited from 40 designated health facilities as part of the parent OPTIC-TB study. Children aged 0–10 years who are diagnosed with TB during the study timeframe and who meet the eligibility criteria will be screened and invited to join at the time of diagnosis or when treatment begins. Eligible children whose caregivers provide written informed consent will be included in the main study. This study will encompass all participants enrolled in the parent OPTIC-TB study in Tanzania. The minimum sample size required for this study is estimated at 379 participants on the basis of the largest sample size needed for the four study objectives. However, since the parent OPTIC-TB study aims to enrol approximately 880 participants, this study will use the entire available sample size. This larger sample will increase the accuracy of the prevalence estimates and ensure adequate power for the planned cross-sectional and cohort analyses. Initially, all the enrolled children will participate in the cross-sectional component of the study at baseline. These same participants will then form a prospective cohort and will be monitored throughout their TB treatment to evaluate treatment outcomes. No additional sampling will be conducted for the cohort component. For cohort analyses, participants will be categorised on the basis of their comorbidity burden at baseline (zero, one, two or three comorbidities) to compare treatment outcomes across different groups.

### Study variables

#### Independent variables

The primary independent variables include the presence of conditions such as malnutrition, HIV infection and DM. Malnutrition will be assessed using anthropometric indicators, including weight-for-age, height-for-age and weight-for-height Z scores, on the basis of the WHO child growth standards. HIV status will be obtained from clinical records or confirmed through testing according to national guidelines. DM will be assessed through the measurement of blood glucose levels using point-of-care testing devices.

#### Dependent variables

The primary outcome variables include TB treatment results as outlined by the national TB programme: treatment success (either cure or completion), treatment failure, loss to follow-up and mortality during treatment. Secondary outcome variables include diagnostic delay, defined as the time interval between the onset of symptoms and the initiation of TB treatment.

#### Covariates

Other factors that could affect TB outcomes, such as age, sex, location of residence, socioeconomic status, nutritional condition and the classification of TB as either pulmonary or extrapulmonary, will also be included.

### Quantitative data collection procedures

Data will be collected by trained research assistants using structured questionnaires and clinical data-extraction forms. Caregiver interviews will collect demographic, socioeconomic, symptom-history and healthcare-seeking data. Clinical records, TB registers and laboratory reports will provide the TB diagnostic method, specimen and result, TB site, HIV status, treatment regimen and outcome. Anthropometry will follow WHO standards using calibrated scales and age-appropriate length boards or stadiometers. Point-of-care capillary glucose will be used as a screening test, not as a stand-alone diagnosis of DM. A fasting value of at least 7.0 mmol/L (126 mg/dL), or a random value of at least 11.1 mmol/L (200 mg/dL) in a child with classic symptoms, will trigger referral and confirmatory testing.^14 22^ In an asymptomatic child, an abnormal result must be confirmed on another day using fasting plasma glucose, HbA1c of at least 6.5% (48 mmol/mol) or another appropriate laboratory test.^14 22^ Existing type 1 or type 2 DM will be abstracted from clinical records. Newly confirmed DM will be distinguished from a single raised glucose result. Hyperglycaemia present only during acute illness and not confirmed on repeat testing will be classified as transient or stress hyperglycaemia, not DM. DM type will be based on the treating clinician’s assessment and supporting clinical or laboratory information, not a glucometer reading alone.

#### Qualitative component

Caregivers of children diagnosed with TB and healthcare providers involved in TB care will be recruited purposively to capture diverse settings and experiences. Approximately 20–30 caregivers and 10–15 healthcare providers will be interviewed, with final sample size guided by information power and thematic saturation. Semi-structured interviews will explore care-seeking behaviour, barriers to TB diagnosis, experiences of TB services and challenges in managing comorbidities. Interviews will be conducted privately and audio-recorded with consent. The interview guide is provided as online supplemental file 1.

### Data management

Trained staff at participating facilities will collect all study data electronically using tablets. Clinical and questionnaire data specific to the study will be entered into a secure, web-based Research Electronic Data Capture (REDCap) database, while Open Data Kit (ODK) will facilitate offline baseline data collection when necessary. To ensure data quality, validation checks, including range and logic checks, will be conducted, along with routine reviews for completeness and consistency. Any queries raised by the study data manager will be promptly resolved in collaboration with staff at each participating facility. Each participant will receive a unique study identification number, and personal identifiers will be stored separately from analytical datasets to ensure confidentiality. Access to the electronic database will be password protected and restricted on the basis of user roles. Electronic data, such as qualitative interview recordings and transcripts, will be securely stored with limited access and archived following approved institutional and ethical procedures.

### Statistical analysis

Quantitative data will be analysed using Stata or R. Baseline characteristics and the prevalence of malnutrition, HIV, confirmed DM and transient hyperglycaemia will be reported with 95% CIs. Cross-sectional analyses will be described as associations and will not be interpreted causally because exposure and TB-related changes may be measured at the same time. Reverse causality will be considered explicitly: TB may cause weight loss and stress hyperglycaemia, while established HIV or DM may precede TB. Analyses will therefore distinguish documented pre-existing disease, newly confirmed disease and transient abnormalities; nutritional findings will be interpreted as concurrent status, and sensitivity analyses will use repeated or follow-up measurements where available. Prospective analyses will relate baseline comorbidity status to outcomes observed later during treatment.

For treatment outcomes, modified Poisson regression with robust standard errors will estimate adjusted risk ratios because treatment success is expected to be common. Prespecified confounders derived from the conceptual framework will include age, sex, socioeconomic position, region, facility, TB site and severity, diagnostic category and OPTIC-TB arm. Malnutrition, HIV and confirmed DM will be entered simultaneously when estimating their independent associations, subject to event counts and collinearity. Potential mediators such as diagnostic delay and service utilisation will be excluded from primary total-effect models and examined in secondary models. Interaction terms will explore whether combined comorbidities have non-additive effects. Facility clustering will be handled with robust clustered standard errors or multilevel models as appropriate. Results will be presented as adjusted effect estimates with 95% CIs.

TB diagnostic method will be summarised by facility. Primary analyses will include all eligible children and adjust for bacteriologically confirmed versus clinically diagnosed TB. Prespecified stratified analyses will report estimates within these two diagnostic groups, and a sensitivity analysis restricted to bacteriologically confirmed TB will assess diagnostic misclassification. Diagnostic delay and service-utilisation outcomes will be analysed using regression models appropriate to their distributions. Missing-data patterns will be described; multiple imputation will be considered when data are plausibly missing at random. Model assumptions, collinearity, influential observations and goodness-of-fit will be assessed. Qualitative interviews will be transcribed, translated where necessary and analysed thematically by at least two researchers where feasible. Quantitative and qualitative findings will be integrated through triangulation.

### Quality assurance

Several measures will be implemented to ensure data quality throughout the study. Research assistants will receive standardised training in study procedures, questionnaires will be pilot tested before field implementation and routine supervision will be conducted during data collection. Double data entry, range checks and periodic verification of records against source documents will be used to minimise transcription and entry errors.

### Patient and public involvement

While patients and the public were not involved in designing or writing this protocol, caregivers will take part in qualitative interviews, offering perspectives that will aid in interpreting the study’s findings. Additionally, patients and the public will participate in the dissemination of the findings once the study is completed.

### Ethics and dissemination

#### Ethical review and approval

Ethical approval for the parent OPTIC-TB trial was granted by the National Health Research Ethics Committee (NatHREC) of the National Institute for Medical Research (NIMR), Tanzania, under Ref. NIMR/HQ/R.8a/Vol.IX/4754, dated 14 November 2024. The approval was extended under Ref. NIMR/HQ/R.8c/Vol.I/3195 on 28 February 2026 and is valid until 28 February 2027. Ethical approval for this nested study was granted by the KCMC University Research Ethics Review Committee (KURERC; Research Proposal No. 1617; Ethical Clearance Certificate No. 2807) on 6 March 2026 and is valid until 5 March 2027. Ethics committee contact emails are ethics@nimr.or.tz for NatHREC/NIMR and drcp@kcmcu.ac.tz for KURERC. The qualitative interview guide is provided as online supplemental file 1. Copies of the KCMC University and NIMR ethical clearances are provided as online supplemental files 2 and 3, respectively.

#### Informed consent and assent

Written informed consent will be obtained from the parents or legal guardians of all participating children before enrolment. Caregivers will be provided with information about the purpose of the study, study procedures, potential risks and benefits, confidentiality and their right to decline participation or withdraw at any time without affecting the child’s access to healthcare services. Assent will be sought from children who are developmentally able to provide it, in accordance with applicable ethical requirements. Participation will be entirely voluntary.

#### Confidentiality and data protection

Confidentiality of participant information will be maintained throughout the study. Each participant will be assigned a unique study identification number, and personal identifiers will not be included in analytical datasets. Study records will be stored securely in password-protected databases accessible only to authorised members of the research team. Data collected during the study will be used solely for research purposes.

#### Risks and benefits

The study poses minimal risk to participants. Anthropometric measurements and blood glucose testing will be conducted using standard clinical procedures. Any clinically important abnormal findings identified during study assessments will be referred for appropriate clinical evaluation and management. Participants may benefit indirectly through improved identification of comorbid conditions, including malnutrition, HIV infection and DM.

#### Dissemination of findings

Study findings will be disseminated through peer-reviewed publications, presentations at national and international scientific conferences, policy briefs and engagement with relevant stakeholders, including the National Tuberculosis Programme and health authorities in Tanzania. Findings will also be shared with participating health facilities to support improvements in the diagnosis and management of paediatric TB.

## Supplementary material

10.1136/bmjopen-2026-120133 online supplemental file 1

10.1136/bmjopen-2026-120133 online supplemental file 2

10.1136/bmjopen-2026-120133 online supplemental file 3

## References

[1] World health organization (2024). Global tuberculosis report 2024.

[2] World Health Organization (2022). WHO Operational Handbook on Tuberculosis: Module 5: Management of Tuberculosis in Children and Adolescents.

[3] Marais BJ, Schaaf HS (2010). Childhood tuberculosis: an emerging and previously neglected problem. Infect Dis Clin North Am.

[4] Pai M, Nicol MP, Boehme CC (2016). Tuberculosis Diagnostics: State of the Art and Future Directions. Microbiol Spectr.

[5] Lawn SD, Zumla AI (2011). Tuberculosis. Lancet.

[6] Lönnroth K, Williams BG, Cegielski P (2010). A consistent log-linear relationship between tuberculosis incidence and body mass index. Int J Epidemiol.

[7] Chiang SS, Khan FA, Milstein MB (2014). Treatment outcomes of childhood tuberculous meningitis: a systematic review and meta-analysis. Lancet Infect Dis.

[8] Venturini E, Turkova A, Chiappini E (2014). Tuberculosis and HIV co-infection in children. BMC Infect Dis.

[9] Joint United Nations Programme on HIV/AIDS. Global HIV Statistics (2022). Global HIV statistics.

[10] Dooley KE, Chaisson RE (2009). Tuberculosis and diabetes mellitus: convergence of two epidemics. Lancet Infect Dis.

[11] Jeon CY, Murray MB (2008). Diabetes mellitus increases the risk of active tuberculosis: a systematic review of 13 observational studies. PLoS Med.

[12] Baker MA, Harries AD, Jeon CY (2011). The impact of diabetes on tuberculosis treatment outcomes: a systematic review. BMC Med.

[13] Harries AD, Kumar AMV, Satyanarayana S (2015). Diabetes mellitus and tuberculosis: public health implications. Lancet Diabetes Endocrinol.

[14] Libman I, Haynes A, Lyons S (2022). ISPAD Clinical Practice Consensus Guidelines 2022: Definition, epidemiology, and classification of diabetes in children and adolescents. Pediatr Diabetes.

[15] Majaliwa ES, Muze K, Godfrey E (2023). Latent tuberculosis in children and youth with type 1 diabetes mellitus in Dar es Salaam, Tanzania: a cross section survey. BMC Infect Dis.

[16] Cochran WG (1977). Sampling Techniques.

[17] Lwanga SK, Lemeshow S (1991). Sample Size Determination in Health Studies: a Practical Manual.

[18] Lönnroth K, Jaramillo E, Williams BG (2009). Drivers of tuberculosis epidemics: the role of risk factors and social determinants. Soc Sci Med.

[19] Storla DG, Yimer S, Bjune GA (2008). A systematic review of delay in the diagnosis and treatment of tuberculosis. BMC Public Health.

[20] Sreeramareddy CT, Panduru KV, Menten J (2009). Time delays in diagnosis of pulmonary tuberculosis: a systematic review of literature. BMC Infect Dis.

[21] Yuen CM, Jenkins HE, Rodriguez CA (2015). Barriers to the diagnosis and treatment of tuberculosis infection and disease in children. Int J Tuberc Lung Dis.

[22] World Health Organization (2019). Classification of diabetes mellitus.

