# Supplementary material for: Magnitude and effects of diabetes mellitus, malnutrition and HIV comorbidities on the management and outcomes of paediatric tuberculosis in Dar es Salaam and Pwani, Tanzania: a mixed-methods protocol
Source: BMJ Open. 2026 Oct 29;16(10):e120133. doi: 10.1136/bmjopen-2026-120133 (PMC13723815; doi:10.1136/bmjopen-2026-120133)
Supplement: Supplementary file 1 [file bmjopen-16-10-s002.pdf]

KURERC 07

KCMC UNIVERSITY

RESEARCH ETHICAL CLEARANCE CERTIFICATE

No. 2807

Research Proposal No. 1617

Study Title: The magnitude and effect of comorbidities of Diabetes Mellitus, Malnutrition and HIV in Management of Paediatric TB in Dar es Salaam and Pwani Regions

Study Area: Dar es Salaam and Pwani Regions

PI's Name: Theresia W. Mbuli

Coinvestigator(s): Blandina Mmbaga, Sayoki Mfinanga and Kushik Ramaiya

Institution (s): KCMC University

The Proposal was approved by KURERC on: 06th March, 2026

Duration of Study: One year

From: 06th March, 2026 to 05th March, 2027

DR. ELINGARAMI. S. NKYA
Chair – KURERC

PROF. EPHATA KAAYA
Vice Chancellor

CamScanner
