# Supplementary material for: Magnitude and effects of diabetes mellitus, malnutrition and HIV comorbidities on the management and outcomes of paediatric tuberculosis in Dar es Salaam and Pwani, Tanzania: a mixed-methods protocol
Source: BMJ Open. 2026 Oct 29;16(10):e120133. doi: 10.1136/bmjopen-2026-120133 (PMC13723815; doi:10.1136/bmjopen-2026-120133)
Supplement: Supplementary file 2 [file bmjopen-16-10-s003.pdf]

THE UNITED REPUBLIC OF TANZANIA

Permanent Secretary
Ministry of Health
Government City Mtumba
Health Road
P.O. Box 743
40478 Dodoma

National Institute
for Medical
Research
3 Barack Obama
Drive
P.O. Box 9653
11101 Dar es
Salaam
Tel: 255 22
2121400
Fax: 255 22
2121360
E-mail:
ethics@nimr.or.tz

NIMR/HQ/R.8c/Vol.I/3195

Prof. Sayoki Mfinanga
National Institute for Medical Research - Muhimbili Medical Research Centre
P. O. Box 3436
Dar es Salaam Region

28 February 2026

RE: APPROVAL FOR EXTENSION OF ETHICAL CLEARANCE

This letter is to confirm that your application for an extension of the study entitled **"Optimizing the implementation and scale-up of the WHO treatment Decision algorithms for children with pulmonary tuberculosis in Sub Saharan Africa OPTIC TB" (Mfinanga, S.** ***et al.*****),** has been approved.

Approval of the extension is based on the progress report dated 17 February 2026 for the project with Ref. NIMR/HQ/R.8a/Vol.IX/4754, dated 14 November 2024. The extension is valid until 28 February 2027.

The Principal Investigator (PI) must ensure that other conditions of approval remain as per the ethical clearance certificate. The PI should ensure that progress and final reports are submitted in a timely manner.

Digitally Signed By Prof. Said S. Aboud
Wed Mar 04 12:17:41 EAT 2026

Prof. Said S. Aboud

CHAIRPERSON
MEDICAL RESEARCH
COORDINATING COMMITTEE

Digitally Signed By Grace Magembe
Fri Mar 13 14:36:36 EAT 2026

Grace Magembe

CHIEF MEDICAL OFFICER
MINISTRY OF HEALTH

CC:Director, Health Services-TAMISEMI, Dodoma.
RMO of Dar es Salaam and Pwani Regions
DMO/DED of respective districts
