# Supplementary material for: Magnitude and effects of diabetes mellitus, malnutrition and HIV comorbidities on the management and outcomes of paediatric tuberculosis in Dar es Salaam and Pwani, Tanzania: a mixed-methods protocol
Source: BMJ Open. 2026 Oct 29;16(10):e120133. doi: 10.1136/bmjopen-2026-120133 (PMC13723815; doi:10.1136/bmjopen-2026-120133)
Supplement: Supplementary file 3 [file bmjopen-2026-120133.reviewer_comments.pdf]

PEER REVIEW HISTORY

BMJ Open publishes all reviews undertaken for accepted manuscripts. Reviewers are asked to complete a checklist review form and are provided with free text boxes to elaborate on their assessment. These free text comments are reproduced below.

ARTICLE DETAILS

Title (Provisional)

Magnitude and Effects of Diabetes Mellitus, Malnutrition and HIV Comorbidities on the Management and Outcomes of Paediatric Tuberculosis in Dar es Salaam and Pwani, Tanzania: A Mixed-Methods Protocol

Authors

Mbuli, Theresia William; Mmbaga, Blandina; Ramaiya, Kaushik; Mfinanga, Sayoki G

VERSION 1 - REVIEW

**Reviewer**	**1**
**Name**	**Sana, Hamaiyal**
**Affiliation**	**Harvard T H Chan School of Public Health**
**Date**	**11-May-2026**
**COI**	**None**

The manuscript is generally well structured. Thank you for sharing it. I enjoyed reading it and found this protocol to be informative for meaningful research and development in TB outcomes research in the pediatric population. However, several areas listed below require further clarification and strengthening:

1. The inclusion of diabetes mellitus as a major comorbidity in pediatric TB patients aged ≤10 years requires stronger justification. The authors should provide more pediatric-specific literature supporting the inclusion of diabetes mellitus, including prevalence estimates.

a. Study should also clarify where type 1 diabetes, type 2 diabetes, or stress hyperglycemia are being considered.

b. If stress hypoglycemia is not being considered, how will the authors differentiate from DBM?

c. Share cut-off values of POC blood glucose measurements.

2. Describe in detail the study limitations in establishing causation with a cross-sectional study component. The study should also consider explaining how reverse causality will be taken into consideration for reporting outcomes, i.e., stress hyperglycemia due to TB, or malnutrition due to TB.

3. How will the authors account for confounding of poor outcomes to attribute to malnutrition, diabetes or HIV? Explain clearly the strategy here. The conceptual framework needs to be more strengthened.

4. Study should provide more details into Tb diagnosis methods between the sites, and also consider doing stratified analysis for patients with confirmed diagnosis and those diagnosed through clinical scoring systems.

5. Please explain your rationale why patients over 10 years were excluded from the study population. Especially considering the increasing burden of adolescent TB and metabolic disease.

6. Consider presenting the analytical observations in a table including the sample size calculations, effect sizes, and prevalence estimates.

7. The study should use more updated references where possible, ideally after 2010.

Overall, this is a useful research question that can benefit ongoing work in the field of TB and comorbidities in pediatric populations. The protocol needs to be further developed methodologically to allow for internal validation and reproducibility.

**Reviewer**	**2**
**Name**	**Kitua, Andrew**
**Affiliation**	**Hubert Kairuki Memorial University, Community Medicine**
**Date**	**17-Jul-2026**
**COI**	**None**

This is a well written protocol for the implementation of a study targeting a very important, but neglected aspect of Pediatric Tuberculosis. All key aspects of the study including background and rationale, objectives, and methodology are well described. The protocol is written with sufficient scientific clarity and the quality of the methods is high.

VERSION 1 - AUTHOR RESPONSE

. Reviewer 1 Comments

No.	Reviewer's Comment	Response	Location in Revised Manuscript
1	The inclusion of diabetes mellitus as a major comorbidity in pediatric TB patients aged ≤10 years requires stronger justification.	Thank you for this important comment. We have strengthened the rationale for including diabetes mellitus (DM) in children aged ≤10 years in the final paragraph of the Introduction. The revised text acknowledges that most evidence on the TB–DM relationship is derived from	Introduction; Line 121 to 132

		adult populations and that paediatric evidence, particularly from sub-Saharan Africa, remains limited. We further clarify that type 1 DM can occur throughout childhood, while type 2 DM is increasingly recognised in younger populations, although it remains less common in children aged ≤10 years. We have also incorporated locally relevant evidence from Dar es Salaam demonstrating a measurable burden of TB infection among children and young people with type 1 DM. Importantly, the study does not presume a high prevalence of DM in this age group; rather, its inclusion is intended to quantify an under-characterised but potentially clinically important comorbidity and to distinguish confirmed DM from transient hyperglycaemia associated with acute TB illness. Relevant references have been cited to support these statements.	
2	Clarify whether type 1 diabetes, type 2 diabetes, or stress hyperglycaemia are being considered.	We now distinguish established type 1 DM, established type 2 DM, newly confirmed DM and transient/stress hyperglycaemia. DM type will be abstracted from clinical records or assigned by the treating clinician using supporting clinical/laboratory information; it will not be inferred from a single glucometer result.	Methods: Quantitative data collection procedures. lines 318–336
3	If stress hyperglycaemia is not being considered, explain how it will be differentiated from diabetes mellitus.	We interpreted 'stress hypoglycaemia' as stress hyperglycaemia. Point-of-care glucose is now explicitly a screening test. A raised value during acute TB illness that is not confirmed on repeat fasting plasma glucose, HbA1c or another appropriate laboratory test will be classified as transient/stress hyperglycaemia and not DM.	Methods: Quantitative data collection procedures; Statistical analysis
4	Provide the cut-off values for point-of-care blood glucose measurements.	The protocol now specifies fasting glucose ≥7.0 mmol/L (126 mg/dL) and random glucose ≥11.1 mmol/L (200 mg/dL) with classic symptoms as thresholds requiring clinical assessment. In asymptomatic children, diagnosis requires confirmation on another day; HbA1c ≥6.5% (48 mmol/mol) is also stated as a confirmatory criterion. A single point-of-care result alone will not diagnose DM.	Methods: Quantitative data collection procedures
5	Describe the limitations of establishing causation with the cross-sectional component and discuss reverse causality.	We expanded the limitations and analysis sections. Cross-sectional results will be reported as prevalence and associations, without causal language. We explicitly recognise that TB may cause weight loss and transient hyperglycaemia. Pre-existing, newly confirmed and transient conditions will be separated, and the prospective cohort will ensure that baseline comorbidity measurement precedes treatment outcomes.	Strengths and limitations; Conceptual framework; Statistical analysis
6	Explain how confounding by malnutrition, diabetes, and HIV will be addressed. Strengthen the conceptual framework.	The conceptual framework now separates exposures, confounders, mediators and outcomes. Primary models will adjust for prespecified confounders including age, sex, socioeconomic position, region, facility, TB site/severity, diagnostic category and OPTIC-TB arm. Malnutrition, HIV and confirmed DM will be mutually adjusted where event counts permit. Diagnostic delay and service utilisation will be treated as potential mediators and excluded from primary total-effect models; interactions and facility clustering will also be assessed.	Conceptual framework; Statistical analysis

7	Provide more details on TB diagnosis methods across study sites and consider stratified analyses by diagnostic method.	We added operational definitions for bacteriologically confirmed and clinically diagnosed TB. The tests, specimen, result and basis of diagnosis will be recorded for every child, allowing site variation to be described. Analyses will adjust for diagnostic category, report strata for confirmed and clinically diagnosed TB, and include a sensitivity analysis restricted to bacteriologically confirmed disease.	Methods: Eligibility criteria; Quantitative data collection; Statistical analysis
8	Explain the rationale for excluding participants older than 10 years.	The age limit is inherited from the parent OPTIC-TB protocol, whose recruitment frame, diagnostic pathway and assessment tools were designed for children aged 0–10 years. This preserves protocol consistency and limits heterogeneity, but we now acknowledge reduced generalisability to adolescents and the need for adolescent-specific research.	Methods: Study population; Strengths and limitations
9	Present the analytical assumptions (sample size, effect sizes, prevalence estimates) in a summary table.	A new summary table presents each quantitative objective, comparison or parameter, prevalence/response assumptions, effect or precision target, attrition allowance and required sample size.	Methods: Sample Size Determination, Table 1
10	Update references where possible, preferably with studies published after 2010.	We updated the supporting literature with recent WHO child and adolescent TB guidance, the WHO Global Tuberculosis Report, current paediatric diabetes guidance and paediatric/East African evidence. Older references were retained only where they are foundational methods or seminal evidence.	Introduction; Methods; References 36–41

C. Reviewer 2 Comments

No.	Reviewer's Comment	Response	Location in Revised Manuscript
1	This is a well-written protocol addressing an important but neglected aspect of paediatric tuberculosis. The background, objectives, and methodology are clearly described, and the quality of the methods is high.	We thank the reviewer for the positive assessment of the protocol and for recognising the importance of studying neglected comorbidities in paediatric TB.	
