# Supplementary material for: Magnitude and effects of diabetes mellitus, malnutrition and HIV comorbidities on the management and outcomes of paediatric tuberculosis in Dar es Salaam and Pwani, Tanzania: a mixed-methods protocol
Source: BMJ Open. 2026 Oct 29;16(10):e120133. doi: 10.1136/bmjopen-2026-120133 (PMC13723815; doi:10.1136/bmjopen-2026-120133)
Supplement: Supplementary file 4 [file bmjopen-16-10-s001.docx]

**Supplementary File 1
Semi-Structured Qualitative Interview Guide**

**Magnitude and Effects of Diabetes Mellitus, Malnutrition and HIV Comorbidities on the Management and Outcomes of Paediatric Tuberculosis in Dar es Salaam and Pwani, Tanzania**

*For caregiver in-depth interviews (IDIs) and healthcare-provider key informant interviews (KIIs)*

**This guide is for adult participants only. Children will not be interviewed as part of the qualitative component.**

# Interviewer instructions

The purpose of these interviews is to explore experiences and perspectives related to paediatric TB diagnosis, service utilisation, treatment and management when TB coexists with diabetes mellitus (DM), malnutrition and/or HIV. Caregiver interviews will be conducted as IDIs, while healthcare-provider interviews will be conducted as KIIs. Interviews should take approximately 45–60 minutes.

Before starting, confirm written informed consent, explain voluntary participation and confidentiality, and obtain permission for audio-recording. Use the participant's study identification number rather than their name. Ask one core question at a time, allow the participant to respond freely, and use probes only when clarification or additional depth is needed. Probes are optional and should not be read as response options.

**Interview identification:** Study ID: __________	Participant: Caregiver / Healthcare provider	Facility: __________	Region: Dar es Salaam / Pwani	Date: __________	Interviewer code: __________

# Part A. Caregiver In-Depth Interview

**A1. Tell me the story of what happened from the time you first noticed that your child was unwell until TB was diagnosed.**

*Optional probes:* What symptoms did you first notice? What did you do before reaching a health facility, and who influenced that decision?

**A2. What, if anything, made it difficult for your child to receive a TB diagnosis quickly?**

*Optional probes:* Were there delays related to transport, cost, referrals, waiting times, repeated visits or availability of tests? Did HIV, diabetes or malnutrition make the diagnosis more difficult or change what happened?

**A3. Please describe your experience of accessing TB services and continuing to attend appointments for your child.**

*Optional probes:* What made attending appointments easier or harder? Were any visits missed or delayed, and what were the reasons?

**A4. What has it been like caring for your child when TB occurs together with HIV, diabetes and/or malnutrition?**

*Optional probes:* How have multiple medicines, clinic visits, feeding needs, tests or costs affected your family? Which aspect of caring for more than one condition has been most difficult?

**A5. Tell me about your child's experience since starting TB treatment.**

*Optional probes:* What changes in symptoms, appetite, weight, activity or general health have you noticed? Have there been side effects, difficulties giving medicines, or missed doses?

**A6. How well have the different services involved in your child's care worked together?**

*Optional probes:* Were TB, HIV, nutrition and diabetes services available in the same place or on the same day? Did you experience problems with referrals, communication between clinics or repeated visits?

**A7. What kinds of support have helped you most while caring for your child?**

*Optional probes:* Consider support from healthcare workers, family members, community members, transport assistance, counselling or nutrition support. What important support was missing?

**A8. Based on your experience, what could be done to improve care for children with TB and other health conditions?**

*Optional probes:* What would make diagnosis, treatment or clinic attendance easier? What should health facilities do differently for caregivers of children with multiple conditions?

# Part B. Healthcare-Provider Key Informant Interview

**B1. Please describe how a child with suspected TB is assessed and diagnosed at this facility.**

*Optional probes:* Which diagnostic tests and referral pathways are commonly used? At which points in the pathway do delays most often occur?

**B2. In your experience, what are the main factors contributing to delays in diagnosing paediatric TB?**

*Optional probes:* How important are caregiver-related, facility-related, laboratory or referral factors? Do these delays differ for children with HIV, diabetes or malnutrition?

**B3. How do HIV, diabetes and malnutrition influence the diagnosis and management of TB in children?**

*Optional probes:* Do these conditions change investigations, monitoring, referral or treatment decisions? What diagnostic or clinical challenges do they create?

**B4. What factors influence whether children and caregivers attend and remain engaged with TB services?**

*Optional probes:* What contributes to missed appointments, delayed follow-up or loss to follow-up? What strategies have helped improve attendance and adherence?

**B5. From your experience, how do DM, malnutrition and HIV affect TB treatment and recovery in children?**

*Optional probes:* What challenges arise with adherence, adverse effects, nutrition, monitoring or treatment response? Are some comorbidity patterns associated with greater clinical or follow-up challenges?

**B6. How are TB services coordinated with HIV, nutrition and diabetes care at this facility?**

*Optional probes:* What currently works well in referral, communication or shared follow-up? Where are the main gaps in integrated care?

**B7. What resources or health-system factors most affect your ability to provide timely and effective care for these children?**

*Optional probes:* Consider staffing, training, diagnostics, medicines, clinic organisation or referral systems. Which resource gaps have the greatest effect on care?

**B8. What changes would most improve diagnosis, service utilisation and treatment outcomes for children with TB and comorbidities?**

*Optional probes:* Which changes are realistic at facility level? Which changes would require district, regional or national programme support?

# Closing question

Is there anything else about paediatric TB diagnosis, treatment, service use or the management of comorbidities that you think is important and that we have not discussed?

Thank the participant for their time and contribution.

# Interviewer field/reflexivity memo

Complete immediately after the interview.
Interview type: IDI / KII
Key issues or themes raised: _________________________________________________
Contextual factors or interruptions: __________________________________________
Participant comfort, sensitivity or hesitation noted: ____________________________
Important non-verbal observations: ___________________________________________
Contradictions or points requiring clarification: ________________________________
Emerging issues to explore in subsequent interviews: ____________________________
